# Incorporating Geographic Information Science and Technology in Response to the COVID-19 Pandemic

**DOI:** 10.5888/pcd17.200246

**Published:** 2020-07-09

**Authors:** Charlotte D. Smith, Jeremy Mennis

**Affiliations:** 1 University of California, Berkeley, School of Public Health, Berkeley, California; 2 Temple University, Philadelphia, Pennsylvania

## Abstract

Incorporating geographic information science and technology (GIS&T) into COVID-19 pandemic surveillance, modeling, and response enhances understanding and control of the disease. Applications of GIS&T include 1) developing spatial data infrastructures for surveillance and data sharing, 2) incorporating mobility data in infectious disease forecasting, 3) using geospatial technologies for digital contact tracing, 4) integrating geographic data in COVID-19 modeling, 5) investigating geographic social vulnerabilities and health disparities, and 6) communicating the status of the disease or status of facilities for return-to-normal operations. Locations and availability of personal protective equipment, ventilators, hospital beds, and other items can be optimized with the use of GIS&T. Challenges include protection of individual privacy and civil liberties and closer collaboration among the fields of geography, medicine, public health, and public policy.

**Summary**

**What is already known about this topic?**

Incorporating geographic information science and technology (GIS&T) into COVID-19 pandemic surveillance, modeling, and response enhances understanding and control of the disease.

**What is added by this report?**

Applications of GIS&T include developing spatial data infrastructures for surveillance and data sharing, incorporating mobility data in infectious disease forecasting, using geospatial technologies for digital contact tracing, integrating geographic data in COVID-19 modeling, investigating geographic social vulnerabilities and health disparities, and communicating the status of the disease or status of facilities for return-to-normal operations.

**What are the implications for public health practice?**

Protections for individual privacy and close collaboration among the fields of geography, medicine, public health, and public policy to use GIS&T are imperative.

## Introduction

The spread of infectious disease is inherently a spatial process; therefore, geospatial data, technologies, and analytical methods play a critical role in understanding and responding to the coronavirus disease 2019 (COVID-19) pandemic. Geographic information science and technology (GIS&T) is the academic field centered on geospatial data and analysis. The field encompasses geographic information systems (GIS), spatial statistics and visualization, and location-based data derived from global navigation satellite systems (GNSS, eg, global positioning systems [GPS]) and remotely sensed imagery. Opportunities for incorporating GIS&T into COVID-19 pandemic surveillance, modeling, and response include 1) developing spatial data infrastructures (SDI) for surveillance and data sharing, 2) incorporating mobility data in infectious disease forecasting, 3) using geospatial technologies for digital contact tracing, 4) integrating geographic data in COVID-19 modeling, 5) investigating geographic health disparities and social vulnerabilities, and 6) communicating the status of the disease or status of facilities for return-to-normal operations. Locations and availability of personal protective equipment, ventilators, hospital beds, and other items can be optimized with the use of GIS&T.

## Developing Spatial Data Infrastructures for COVID-19 Surveillance and Data Sharing

Current surveillance of COVID-19 at the national and global levels is built on lessons learned from maintaining previously developed databases of contamination and disease, such as FluNet (1). Disease surveillance systems have been enhanced by the use of GIS for monitoring disease outbreaks, facilitating contact tracing, and evaluating the efficacy of interventions. For example, Zenilman et al described the application of GIS to a surveillance system for sexually transmitted diseases at the Fort Bragg military base (2). The assessment of various potential risk factors indicated that geography was the only variable positively associated with gonorrhea among study participants. The Connect to Protect program is an example of how researcher–community collaborations (or community-based participatory research) can assist program planners to efficiently use limited resources (3). Connect to Protect, a nongovernmental organization, uses GIS and community involvement to prioritize resources and outreach activities. The research team uses GIS to evaluate the geographic epidemiology of sexually transmitted diseases and HIV among adolescents in 15 US cities and Puerto Rico. Their work led to a shift from traditional evaluations of condom use, number of sex partners, and demographic characteristics, to identification of sociophysical environments. The observation of clusters of cases in geographic areas informed research teams on where to apply interventions. The use of GIS supports the investigation of the social and environmental correlates of disease clusters, thereby facilitating targeted interventions and researcher–community collaborations to assist program planners to efficiently use limited resources (3).

An important aspect of monitoring the spread of infectious disease is spatial data infrastructure (SDI), composed of the human resources and institutions that create and maintain the foundation to which additional spatial data can be attached and used. Key components of an SDI include geospatial culture and awareness, resources for information and communications technology, common standards for data integration and interoperability, a legal framework for data security and privacy, a common lexicon, the use of robust statistical and epidemiological methods, and interdisciplinary collaboration and partnerships (4). Along with the SDI, the concepts of open data, crowd sourcing, and data sharing for georeferenced health data are important components of real-time infectious disease surveillance, particularly in under-resourced settings (5).

Maps play a key role in communicating the risks and spread of COVID-19 (6). Interactive web-based maps and dashboards present near–real-time data on morbidity, mortality, and recovery, as well as pandemic-related factors such as supply-chain quantities of personal protective equipment or therapeutics. A dashboard developed by Johns Hopkins University in collaboration with ESRI (Redlands, California), which originally showed the number of COVID-19 cases, deaths, and recoveries, was updated to show smaller geographic areas (ie, counties) and detailed infographics (7). This type of infographic has been useful for tracking COVID-19 cases globally and for allocating resources and planning for “return-to-normal” conditions. Location-enabled infographics also allow for dissemination of knowledge on, for example, the readiness of facilities such as retail outlets to accept customers, or schools and campuses to reopen. An interactive dashboard (ESRI, Redlands, California), developed for faculty, staff, students, and administrators at the University of California, Berkeley, shows the status of custodians’ efforts to disinfect university buildings (Figure). The dashboard is populated in real time as custodial staff members complete disinfection of rooms. The room number and type (eg, classroom, laboratory, bathroom), the date and time completed, and the product used for disinfection appear in a pop-up on the dashboard when the user selects a building.

**Figure:**
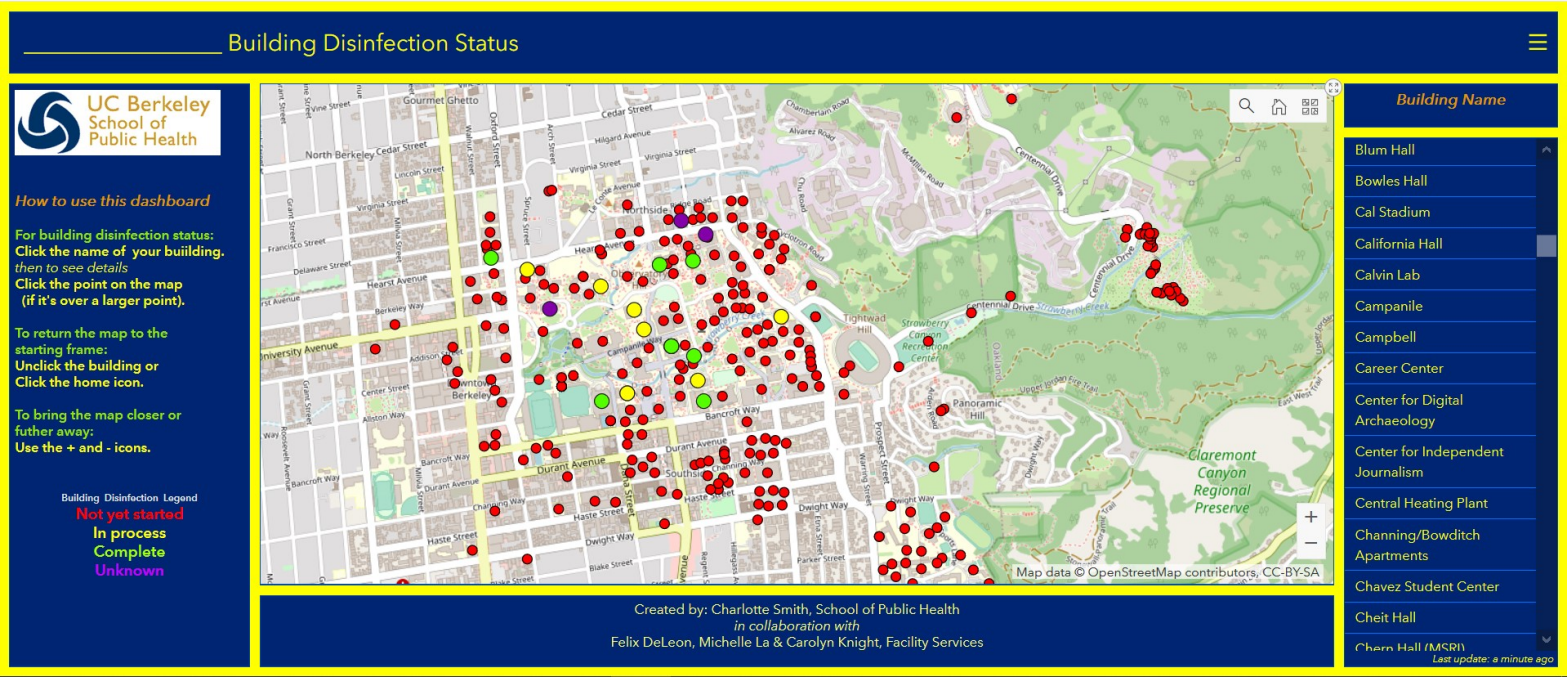
An interactive dashboard for showing the status of disinfection of buildings during the coronavirus disease 2019 (COVID-19) pandemic on the campus of the University of California, Berkeley.

The GIS&T community has long worked toward development of the National Spatial Data Infrastructure (NSDI) for the United States (8), an effort managed by the US Federal Geographic Data Committee (FGDC); facilitated by spatial data interoperability standards, such as those developed by the Open Geospatial Consortium (OGC); and recently bolstered by the Geospatial Data Act of 2018, a component of H.R.302, the FAA Reauthorization Act of 2018. The US NSDI is typically considered an infrastructure for geospatial framework data (eg, cadastral and transportation) and not necessarily health data; however, just as the events of September 11, 2001, catalyzed the development of enhanced spatial data sharing to support disaster response in the United States, the COVID-19 pandemic has the potential to spark the improvement of health data infrastructures to facilitate spatial data sharing and interoperability for health crisis response. A particular challenge is that SDIs for responding to a crisis like COVID-19 require sharing data not only among various national and international governments but, as with the US NSDI, also among various levels of government, including the federal, state, and county levels. Corporate partners also play a pivotal role in the development of SDI for pandemic response, because they have large sets of spatial data on the mobility, purchasing, and web browsing behaviors of individuals and other relevant place-based and georeferenced data that may be useful in understanding disease dynamics. In addition, responding to a rapidly evolving health crisis such as the COVID-19 pandemic requires pipelines for supplying health and related data in near real time, which presents challenges. Finally, privacy protection for individuals is paramount in developing useful SDIs for pandemic response. As with the US NSDI, initiative and management at the federal level is likely necessary to develop an SDI for pandemic response.

## Incorporating Population and Mobility Data in COVID-19 Forecasting

Along with handwashing and social distancing, perhaps the foremost mitigation strategy for reducing person-to-person contact and transmission of severe acute respiratory syndrome coronavirus 2 (SARS-CoV-2) in the absence of pharmaceutical intervention is regulation restricting mobility (ie, human movement and travel behavior). Consequently, one key role for geospatial technologies in responding to the COVID-19 pandemic is monitoring population distribution and mobility through the use of social media and location-tracking applications embedded in mobile telephones that employ GNSS, cell phone tower connections, and/or wireless connections (9). Several corporate location-data collectors and vendors have released spatially aggregated COVID-19 pandemic-related data on population mobility. These data have been widely used by the popular media to report on the effects of jurisdictional stay-at-home orders on population mobility and by researchers to analyze the efficacy of population mobility change for altering disease dynamics (10).

Modeling population distribution and mobility has a long history in GIS&T and focuses on fine-scale estimations of population distribution and mobility (11,12), most recently by using mobile telephone–based location data (13,14). The scholarly response to the pandemic marks a major advance in the incorporation of fine-resolution data on population and individual mobility from geospatial technologies to understand disease dynamics and formulate effective intervention strategies. Because questions remain about the best way to measure and collect data on individual mobility, provide such data to researchers, and incorporate such mobility measures into infectious disease models, the COVID-19 pandemic provides an opportunity for testing methods for using such data to evaluate and forecast the effects of nonpharmaceutical interventions that restrict mobility. However, current legal frameworks and practices for preserving the privacy of individuals are obstacles to widespread adoption.

## Using Geospatial Technologies for Digital Contact Tracing

Monitoring mobility at the individual level, in addition to the population level, has also emerged as an important use of geospatial technologies, particularly in its application to digital contact tracing. Conventional contact tracing, involving identifying, contacting, and encouraging quarantine for the people with whom an infected person has had close contact to mitigate disease transmission, is labor intensive. The process can be made more efficient and scaled up to large populations by exploiting individual digital mobility data, as well as data indicating proximity among mobile telephones using Bluetooth or related technologies, to computationally show close proximity among individuals (15). Such location data can be combined with health and other data that might indicate vulnerability to infection or disease. Individuals can then be contacted and given quarantine instructions automatically through mobile telephone text messages, or their future behavior may even be monitored to encourage or enforce quarantine. Such procedures have been used to some degree, in combination with population mobility restrictions, in an attempt to reduce SARS-CoV-2 transmission in China, Israel, Singapore, and South Korea, among other nations, and developments for digital contact tracing technologies by the largest international technology companies continue (16).

Advances in GIS&T have been made in modeling the geographic trajectories of individuals throughout their daily lives, their interactions with other people, and their immediate environment using geographic and computational constructs such as activity space and space–time prisms (17–20). However, to leverage this body of research for digital contact tracing, progress needs to be made in developing, testing, and implementing digital contact tracing applications, including evaluations of behavioral compliance, efficacy, and scaling. Additionally, this approach raises concerns about confidentiality and civil liberties that need to be addressed before widespread adoption (21).

## Integrating Geographic Data in COVID-19 Modeling

A strength of GIS is the ability to integrate diverse spatial data sets based on georeferencing, facilitating the integration of health data with contextual characteristics. Descriptive modeling research that leverages this capability has examined the spatial associations of COVID-19 with socioeconomic and environmental characteristics. This research found, for example, that lower income and income inequality (22), higher temperature and humidity (23), exposure to fine particulate air pollution (24), and mobility and transportation networks (25,26) were associated with a higher prevalence of COVID-19 cases or mortality. GIS&T also offers approaches to investigating statistical spatial effects and spatial heterogeneity, such as spatial autoregressive models and geographically weighted regression, to account for modeling geographic processes such as spatial diffusion and the variation in relationships among variables over space (27,28). Recent research leveraged these approaches in demonstrating the spatial heterogeneity in the relationships among observed COVID-19 cases and mortality with georeferenced socioeconomic and environmental variables (22,29,30) and found that the influence of area-based socioeconomic status, pre-existing health conditions, and environmental characteristics on disease transmission may vary from place to place.

Computational infectious disease models are widely used to predict or forecast the spread of COVID-19 disease and the effects of intervention strategies. Predictive modeling approaches can be generally categorized as SEIR/SIR (susceptible, exposed, infected, and removed/recovered) (31), agent-based (32), or statistical modeling (33). Such modeling approaches are inherently geographic in the sense that they make predictions for certain areas or regions, although only some models contain an explicit spatial interaction component or forecast the spatial variation in disease incidence over small areas. Explicitly incorporating a spatial component into infectious disease models attempts to account for 1) place-based contextual mechanisms of infection or disease related to the socioeconomic, built, or natural environments, such as air pollution or type of employment, 2) spatial heterogeneity in the drivers of disease transmission, for example, where certain socioeconomic characteristics may be associated with disease prevalence in one region but not in another as a result of regional differences in culture or behavioral norms, and 3) transportation networks or patterns of human mobility to better account for disease transmission dynamics (34,35). Such approaches have been extended to modeling the spread of COVID-19, providing evidence that restrictions on mobility have mitigated the spread of COVID-19 in different parts of the world and aided in forecasts of disease diffusion under various scenarios of mobility restriction (36,37).

Spatial transportation and mobility data can play an important role in forecasting disease prevalence, where, for example, the effect of nonpharmaceutical interventions (eg, restrictions on mobility) on city-level transmission of COVID-19 in China was analyzed using mobility data harvested from mobile telephone location-based services. This method allows one to parameterize the local contact rate and forecast the geographic distribution of disease prevalence under different intervention timing scenarios (37). Related approaches to modeling the spread of COVID-19 also incorporated airline transportation networks (38) and were extended to other countries with extensive COVID-19 outbreaks, such as Italy (36), providing substantial evidence that restrictions on mobility have mitigated the spread of COVID-19 in different parts of the world.

## Investigating Geographic Health Disparities of the COVID-19 Pandemic

Indices of social vulnerability are place-based variables that incorporate factors such as race/ethnicity and socioeconomic status to encode the vulnerability to adverse health outcomes and other types of hazards (39). Community social vulnerability, along with health care resources, plays an important role in predicting health care capacity in responding to the COVID-19 pandemic (40). Social vulnerability can interact with pre-existing medical conditions and access to medical resources, such as prescription drugs, to produce inequities in COVID-19 outcomes (41). People with underlying medical conditions, such as asthma, obesity, and diabetes, as well as people who are immunocompromised or aged 65 or older are at higher risk of serious consequences from SARS-CoV-2 infection than their healthier or younger counterparts. Because such medical conditions are often concentrated geographically and among certain demographic groups, understanding the spatial and demographic distribution of these conditions is critical to investigating health disparities associated with COVID-19. For example, COVID-19 morbidity and mortality are higher among African American and Hispanic people than among non-Hispanic white people (42). Such racial/ethnic disparities highlight the importance of efficient collection of socioeconomic, demographic, and other data among people with COVID-19.

Resources for investigating COVID-19-related social disparities include publicly available data on COVID-19 cases by small areas, such as zip codes (43), although such data are not widely available at a national level. The same issue exists for fine spatial resolution data on social vulnerability. The Public Health Disparities Geocoding Project at the Harvard T.H. Chan School of Public Health seeks to address this latter shortcoming (44). Researchers should understand the geographic and historical background of discrimination and resource deprivation that may produce place-based social vulnerabilities, to avoid stigmatizing or placing blame on certain communities. An understanding of the social determinants and structural forces, such as food insecurity, housing insecurity, and disparities in educational or health care infrastructure, that can influence health outcomes such as obesity, hypertension, and certain types of cancer, is important.

The multidimensional social, economic, and health consequences of the COVID-19 pandemic are geographically inequitable: some places and populations have greater social, economic, health and other effects than other places and populations. Beyond the need to identify such factors as lack of access to resources or the prevalence of pre-existing health conditions is the need to recognize and understand the mechanisms of vulnerability that have been in place and led to the exacerbation of the COVID-19 crisis in some communities. Community recovery from the COVID-19 pandemic requires incorporation of social, economic, and health components and an emphasis on investigating how place shapes the uneven effect of COVID-19.

## Implications for Public Health

We have outlined how GIS&T can be used for understanding and responding to the COVID-19 pandemic and future infectious disease epidemics and pandemics. Central to this understanding and response is a commitment for the use of GIS and geospatial technologies as the platform for collecting, integrating, and analyzing georeferenced data on the locations and characteristics of individuals and the spatial distribution of socioeconomic, health, and built and natural environmental characteristics. Geospatial resources for COVID-19 response are available through several organizations, including the University Consortium for Geographic Information Science (www.ucgis.org/covid-19-resources), the OGC (www.ogc.org/resources-for-COVID-19-from-ogc), and the National Alliance for Public Safety GIS Foundation (www.napsgfoundation.org/resources/covid-19).

Leveraging GIS&T for responding to the COVID-19 pandemic requires a close and extensive collaboration between researchers in the fields of geography, medicine, public health, and public policy. The field of GIS&T has a long history of research in data synthesis, statistical modeling, and computational simulation for spatial data and applications. Recognizing that GIS&T is a theoretical and scientific approach rather than simply a set of analytical tools will facilitate transdisciplinary collaboration. Advances in preserving individual privacy and civil liberties in the age of big spatial data, where geospatial technologies generate massive repositories of individual-level data on movement, health, and behavior widely available, are also necessary. These advances will likely require enhanced government regulations, corporate policies, and technological innovations in data sharing and privacy protection.

The COVID-19 pandemic is still in the beginning phase, and the research community is continuing to learn and revise the best way to respond to this global public health crisis. Geospatial data, methods, and technologies have a crucial role to play in understanding and responding to the pandemic, and the lessons learned on the use of GIS&T for pandemic response at this time should enhance preparedness and response for future public health crises.
